# Proton Pump Inhibitors: Exploring Cardiovascular Complications and Prescription Protocol

**DOI:** 10.7759/cureus.16744

**Published:** 2021-07-29

**Authors:** Mubashira K Sarnaik, Srimy Modi, Yasaswi Pisipati, Sarayoo Vaidya, Naqvi Syed Gaggatur, Aliya H Sange, Natasha Srinivas, Ibrahim Sange

**Affiliations:** 1 Internal Medicine, M S Ramaiah Medical College, Bangalore, IND; 2 Internal Medicine, K. J. Somaiya Medical College, Mumbai, IND; 3 Internal Medicine, Dubai Medical College, Dubai, ARE; 4 Internal Medicine, BGS Global Institute of Medical Sciences, Bangalore, IND; 5 Research, California Institute of Behavioral Neurosciences & Psychology, Fairfield, USA; 6 Medicine, K. J. Somaiya Medical College, Mumbai, IND

**Keywords:** proton pump inhibitors (ppi), cardiovascular side effects, prescription patterns, gastro-intestinal bleed, gastroesophageal reflux disease, clopidogrel

## Abstract

Proton pump inhibitors (PPIs) are among the most extensively prescribed medications internationally for gastroesophageal reflux disease treatment and the prevention of gastrointestinal bleeding. Their efficiency, ease of availability, and low side effect profile offer several advantages over other treatment modalities. Long-term use and inappropriate prescribing habits have increased the presence of this class of drugs, prompting several studies to reassess their adverse effects. This article explored the possibility of a relationship between PPIs and cardiovascular adverse effects while highlighting the current prescription guidelines for PPIs. We further examined the need for more research into the etiology of PPI-related cardiovascular adverse effects and strategies to alleviate these risks.

## Introduction and background

Gastric acid secretion is a vital component of the digestive system. Gastric acid is secreted mainly by parietal cells and serves to sterilize food intake, facilitate its absorption, aid digestion, and provide defense against pathogens [1]. Gastro-protective mechanisms include bicarbonate secretions, intact mucosa lining the gastrointestinal tract, and the sealing effect of the gastroesophageal sphincter contractions [2].

Irregularities in gastric acid secretion occur through two main processes: excess secretion of acid or breaks in the protective mucosal barriers. Interruption in the gastro-protective mechanisms can occur due to emotional stress, nonsteroidal anti-inflammatory drugs (NSAIDs), ethanol, malnutrition, *Helicobacter pylori* infection, or sphincter insufficiencies [2]. 

Dyspepsia is a fairly common condition that encompasses a constellation of clinical features of early satiety, heartburn, regurgitation, and epigastric pain. Dyspepsia can be either functional or due to disorders such as gastroesophageal reflux disease (GERD), *Helicobacter pylori* infection, achalasia, or Barrett's esophagus [2]. The first line of management for these disorders will usually include a drug from the class of proton pump inhibitors (PPIs) [3]. These drugs predominantly block the hydrogen-potassium ATPase pump leading to an inhibition in the release of gastric acid and thus a fall in the gastric pH. These agents are well tolerated in a broad spectrum of patients, but adverse effects of gastritis, nephritis, and bone density loss, to name a few, have been reported [4].

In the United States, due to their effectiveness, easy availability, and low cost, PPIs have become a routine inclusion in the pharmacotherapy practices of physicians today [5]. In 50% of hospitals and ambulatory settings, PPI overuse was prevalent, with inappropriate prescriptions accounting for 50% of PPI usage post-hospital discharge [6]. The indications ranged from prophylactic gastric mucosal protection for drugs not associated with mucosal damage to incorrect gastroesophageal disorder diagnoses [6,7]. 

A conventionally recommended PPI regimen duration ranges from two to eight weeks, extending to 12 weeks, with dosing of once or twice daily depending upon the patients' individual needs [8]. With $13 billion in sales and 113 million annual prescriptions internationally, PPI usage is exponentially growing, especially in the elderly. They were found to use PPIs consistently over extended periods, with a median treatment duration of 1 to 4.6 years [8,9]. The need for analysis into long-term side effects becomes imperative, with PPIs' increasing presence in pharmacotherapy regimens.

Proton pump inhibitors are known to increase the risk for kidney disease, osteoporosis, and infections like pneumonia in the elderly population [10]. Studies have found increased cardiovascular morbidity and mortality in patients taking PPIs and clopidogrel, which prompted the FDA to issue warnings for the combination [11]. Cardiovascular events included myocardial infarction, stroke, transient ischemic attacks, and cardiovascular death, to name a few [12]. Additional research showed that cardiovascular risks differed between different PPIs and were present in patients not on clopidogrel therapy [12]. Further exploration of the possibility of increased cardiovascular complications of PPIs is warranted.

This article aims to:

1. Establish a link between increased cardiovascular complications (myocardial infarction, transient ischemic attacks, and cardiovascular death) and PPI treatment.

2. Highlight existing protocol for addressing increased cardiovascular risks.

3. Identify possible strategies to mitigate these risks and improve PPI treatment regimens.

## Review

Proton pump inhibitors

Proton pump inhibitor use has increased drastically in the last few decades. A study by Muheim et al. revealed that the incidence of PPI prescriptions rose from 19.7% (2012) to 23.0% yearly (2017), of which the incidence of potentially inappropriate PPI prescriptions rose from 4.8% (2013) to 6.4% (2017). Patients with comorbidities and those requiring drugs with a bleeding risk had a propensity for improper use of PPIs [13].

The adverse effects of these drugs should be taken more seriously due to their widespread use. A study in Hungary revealed that the average age of PPI users was 65 years old, with a minimum treatment interval of six months. One-fifth of the population had extended use for more than five years [14]. Patients usually do not self-deprescribe, and most primary care physicians tended to continue the same treatment without reevaluation for the need for PPIs [15].

One reason for the continuation of PPIs was the development of a possible addiction through hyperplasia of enterochromaffin-like cells, which secrete histamine, stimulating the proton pump. Rebound gastric secretion can occur on withdrawal of PPIs due to this effect, leading to extended overuse [16].

PPI and clopidogrel interactions

For many years the possibility of PPI and clopidogrel interaction was a concern, with several studies suggesting that PPIs reduce the activity of clopidogrel. This led to the FDA issuing a warning about the combination in 2009 [11]. Further studies have found that the increase in cardiovascular complications may be due to the PPI rather than the specific interaction between the PPI and clopidogrel [17].

Moayyedi et al. conducted a randomized controlled trial in 2019 by studying 17,598 patients with stable cardiovascular disease and peripheral artery disease for the effect of proton pump inhibitors. The PPI group consisted of 8791 patients. Patients were randomly assigned to a group of antithrombotics, either rivaroxaban (2.5mg twice daily) with aspirin, rivaroxaban only (5mg twice daily), or aspirin only (100mg). Participants were evaluated over three years. On analysis, no statistically significant difference between the PPI group and placebo group was found for cardiovascular outcomes of myocardial infarction (MI), stroke, or cardiovascular death (Table 1) [18]. 

**Table 1:** Summary of studies examining the link between PPI and Clopidogrel concomitant use PPI- proton pump inhibitor; RCT- randomized control trial; bd- twice daily; od- once daily; PAD- peripheral arterial disease; DAPT- dual antiplatelet therapy; ACS- acute coronary syndrome

Study (year)	Design	Total patients (patients taking PPIs)	Patient condition	PPI assigned	Medications assigned	Comments
Moayyedi et al. (2019) [18]	RCT	17,598 (8,791)	Stable cardiovascular disease and PAD	Pantoprazole 40mg daily	Rivaroxaban (2.5mg bd) with Aspirin (100mg od), Rivaroxaban (5mg bd), Aspirin (100mg)	No statistically significant difference between groups
Przespolewski et al. (2018) [19]	RCT	28 (28)	Healthy male	40mg Pantoprazole, 20mg Omeprazole, 20mg Rabeprazole, 40mg Esomeprazole, 30mg Lansoprazole, 30mg Dexlansoprazole	Clopidogrel 75mg daily	Aggregation did not increase significantly with PPI-Clopidogrel use
Vaduganathan et al. (2016) [20]	RCT	Low dose Aspirin-2,480 (1,231), High dose Aspirin-1,272 (638)	Patients requiring DAPT treatment for at least 12 months	Omeprazole 20mg	Clopidogrel 75mg	Randomized PPIs did not affect the cardiovascular outcome of patients
Bhurke et al. (2012) [21]	Retrospective cohort study	5,348 (2,674)	ACS patients with Clopidogrel prescription	Any identified PPI use	Clopidogrel	Increased risk of adverse cardiovascular events with PPI-Clopidogrel use
Juurlink et al. (2009) [22]	Nested case-control study	Controls- 2,057 (424), Cases- 734(194)	Controls- Event-free patients after MI Cases-patients readmitted following an acute MI	Pantoprazole-46/734, Other-148/734	Clopidogrel	PPI-Clopidogrel use showed increased risk in elderly with reinfarction, barring Pantoprazole

**Figure 1:**
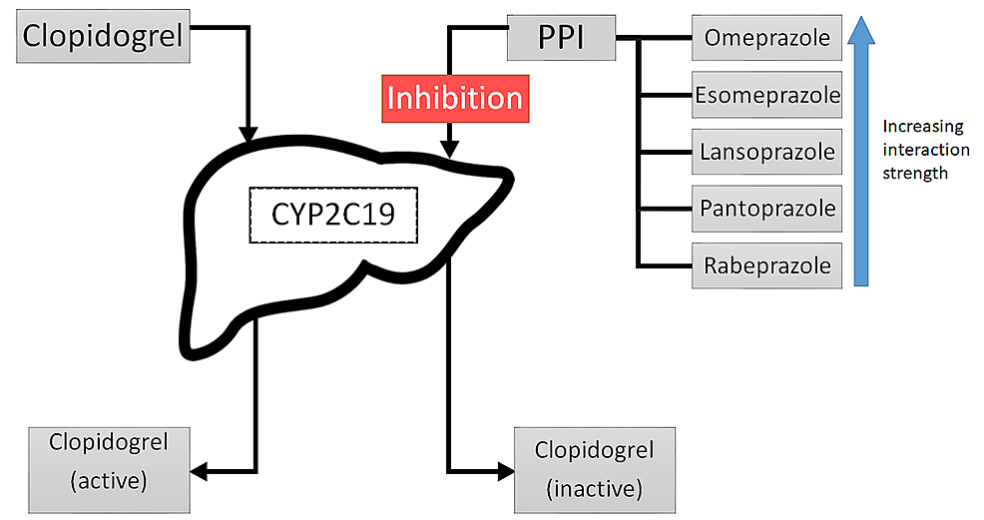
Suggested mechanism of Clopidogrel inactivation through CYP2C19 enzyme by proton pump inhibitors PPI- proton pump inhibitor; CYP2C19- Cytochrome P450 2C19 hepatic enzyme

The possible interaction of proton pump inhibitors with clopidogrel brought forth the question of whether this effect was specific to PPIs (like omeprazole) that function through the inactivation of the CYP2C19 pathway, inhibiting clopidogrel activation. A study was conducted in New York in which 28 healthy males were randomized to receive one of three PPIs and clopidogrel daily, with a one-week clopidogrel washout period. They were subject to an incomplete crossover design schedule, followed by platelet aggregation testing. The study concluded that there was no significant change in aggregation with any PPI but did not rule out a possibility of a weak inhibitory effect on clopidogrel that could be aggravated in patients with severe vascular disease and comorbid conditions [19]. Based on these results, a careful look at the patient profile is warranted while prescribing PPIs to prevent inhibition of clopidogrel (Figure 1).

Considering the comorbid state of a patient, we looked at studies involving different pre-existing cardiovascular conditions to see the impact of PPIs. Based on data collected from the COGENT (Clopidogrel and the Optimization of Gastrointestinal Events Trial), a posthoc analysis was conducted in patients requiring dual antiplatelet therapy (DAPT) treatment for a minimum of 12 months, involving 3752 patients, of which 2480 were assigned to the low dose aspirin group, and 1272 were assigned to the high dose aspirin group based on medication records [20]. COGENT was a global prospective, phase III randomized, double-blind, double-dummy clinical trial in which participants were randomly assigned a fixed combination of omeprazole 20mg with clopidogrel 75mg or clopidogrel 75mg alone [17]. It was found that incidence of significant cardiovascular endpoints did not increase with PPI treatment in either aspirin dosing group, but PPIs were ruled vital for preventing gastrointestinal (GI) bleeding in coronary artery disease patients on DAPT, alongside aspirin treatment. All-cause mortality rates remained low with both subsets. This study supports the general recommendation that the continued use of PPIs outweighs the possible side effects [20].

A retrospective cohort study conducted in 2012 selected a group of acute coronary syndrome (ACS) patients with clopidogrel prescriptions to search for PPI use and evaluate for adverse cardiovascular endpoints. Of the 5348 patients selected, 2674 were identified to have used an overlapping PPI regimen. The selected clopidogrel-PPI group was matched 1:1 with the clopidogrel-only group. Results showed a significantly increased risk of cardiovascular events (HR= 10438; 95% CI, 1.237-1.671) in the clopidogrel-PPI group. This study had the advantage of selecting more geographically diverse populations with a study period spanning eight years. The process of CYP2C19 inhibition was suggested as the reasoning behind possibly inactivated clopidogrel. Considering the large population surveyed over a long period, it further supports that long-term PPI use may have adverse outcomes. Patients with comorbidities were identified to have a greater risk of adverse cardiovascular events [21].

A similar endpoint was seen in a population-based nested case-control study conducted by Juurlink et al. on patients 66 years and above on post-hospital discharge for myocardial infarction treatment. Patients that were readmitted for acute myocardial infarction within 90 days of discharge were selected. Controls were matched at a ratio of 3:1, and PPI exposure was studied. Of 13,636 patients prescribed clopidogrel post-MI, 734 were readmitted for reinfarction, and 2057 controls were assigned. Proton pump inhibitor use was associated with a higher risk of reinfarction, barring pantoprazole use [22].

PPI effect on newly prescribed patients and GERD patients

To understand the impact of PPIs on the general population without comorbidities, we considered studies involving new PPI therapy patients (Table 2). A case-control study was conducted in Italy using the NHS database to identify 17,832 PPI users hospitalized for cardiovascular and cerebrovascular diseases and match them with up to five controls. It was found that there was a significantly increased risk of cardiovascular events requiring hospitalization for current and recent PPI users, irrespective of the type of PPI. Again, this suggests association but not causation of these cardiovascular events. A further point to note was the lack of similar cardiovascular outcomes in the patients taking histamine type 2 receptor antagonists (also referred to as H2 antagonists) [23].

**Table 2:** Summary of studies examining cardiovascular adverse events in gastroesophageal reflux disease patients using proton pump inhibitors PPI- proton pump inhibitors; RCT- randomized controlled trial; GERD- gastroesophageal reflux disease; SOPRAN- Safety of Omeprazole in Peptic Reflux Esophagitis: A Nordic Open Study; LOTUS- Long Term Usage of Esomeprazole vs. Surgery for Treatment of chronic GERD; TIA- transient ischemic attack; MI- myocardial infarction; CV event- cardiovascular event

Study (year)	Design	Total patients (patients taking PPIs)	Patient condition	PPI assigned	Comments
Casula et al. (2018) [23]	Nested case-control study	Control (89,160), Cases (17,832)	New PPI users	any PPI	The risk of a new-onset cardiovascular event was higher in current and recent users
Xie et al. (2017) [24]	Observational cohort study	1,762,908 (275,977)	New PPI users	any PPI	Current and past use of PPIs was associated with increased risk of death
Attwood et al. (2015) [25]	Open-label RCT	SOPRAN: 298 (154), LOTUS: 514 (266)	GERD	SOPRAN: Omeprazole, LOTUS: Esomeprazole	Omeprazole group: CV events- 28, TIA events- 3, Esomeprazole group: MI events- 5, angina events- 3
Lundell et al. (2009) [26]	RCT	310 (154)	GERD	Omeprazole	PPI group: death due to heart complications- 8, Non-fatal MI event- 9
Lundell et al. (2008) [27]	RCT	554 (266)	GERD	Esomeprazole	CV event- 3 in the PPI group; CV event- 4 in the surgery group

Xie et al. conducted an observational cohort study to identify the association of PPI with all-cause mortality, utilizing information from the United States Department of Veteran Affairs database. Based on a cohort of 1,762,908 patients, 275,977 patients were identified as new PPI users and included in the first cohort. They compared new PPI users to H2 antagonist users. Secondary studies included new PPI users compared to non-PPI users. A further comparison with non-PPI and non-H2 antagonist users was made. The study found that PPI users had higher risks of all-cause mortality in each scenario. Those without gastrointestinal complaints and more extended use were identified to have a higher risk. The limitations of this study were that cause of mortality was not identified for further analysis, and the population studied consisted of mostly older, white males, restricting the generalizability of the results [24].

Since many studies have been carried out in patients with pre-existing cardiovascular disease to assess PPI effects, we took a look at studies in patients with gastroesophageal reflux disease to see whether the risk of cardiovascular adverse effects could be discerned.

Attwood et al. analyzed data collected from the SOPRAN and LOTUS clinical trials to study the long-term effects of PPI therapy. Myocardial infarction, angina, and cardiac failure events were more common with omeprazole than the open antireflux surgery (ARS) group in the SOPRAN trial. The MI events were ruled inconclusive based on prior patient history after further analysis by the FDA of trial data. Cardiac failure was seen more in the esomeprazole group in the LOTUS trials. It was determined that more data would be needed to derive a conclusion on cardiovascular events [25].

Lundell et al. conducted a study to analyze the long-term effects of GERD therapies over 12 years, involving the patients of the SOPRAN trial, posited similar conclusions on the MI incidence in omeprazole trials [26]. Lundell et al. also led a three-year interim analysis of the LOTUS trial with cardiac events occurring in similar amounts in both the esomeprazole and laparoscopic antireflux surgery groups [27]. The conclusion was that PPIs offered effective control of GERD and remained safe and well-tolerated [26,27].

One study conducted data mining to collect over 16 million clinical documents for 2.9 million individuals to study the risk of cardiovascular complications in the general population. The results showed that gastroesophageal reflux disease patients exposed to PPIs had 1.16 times increased association with myocardial infarction. Additionally, a two-fold increase in association with cardiovascular mortality, irrespective of clopidogrel use, was found. They concluded that the risks of PPIs extend beyond high-risk patients to involve the general population. However, they could not draw conclusions based on different doses of PPIs and over-the-counter use [9].

Existing guidelines for PPI prescription practices

The American Gastroenterological Association guidelines (2017) [28] state that:

1. Patients with GERD should be prescribed short-term PPI therapy for healing and maintenance.

2. On the resolution of complaints, PPI therapy must be reduced or stopped. If a reduction is not possible, patients can consider ambulatory esophageal ph/impedance monitoring to assess functional syndromes versus GERD.

3. Barret's esophagus and symptomatic GERD patients can consider long-term PPI, even if asymptomatic. Nonsteroidal anti-inflammatory drug (NSAID) users at risk of bleed may also consider PPI use.

4. Periodic reevaluation of PPI dose in long-term therapy should be done to adjust optimal dosing.

The American Heart Association (AHA) guidelines (2016) have stated that PPIs should be used in patients on DAPT with a history of previous GI bleeding, patients with increased risk of GI bleeding, and those with concomitant use of NSAIDs, warfarin, or steroids [29].

The American College of Cardiology Foundation task force developed a document in 2010 with the American College of Gastroenterology and the AHA for management guidelines for gastrointestinal risks of antiplatelet and NSAID therapy. They recommended PPI therapy for patients with a history of upper GI bleed and patients on antiplatelet therapy with risks for GI bleed. Routine use of PPI or H2 antagonists was not recommended in low-risk patients as prophylaxis [30]. Both cardiovascular and gastrointestinal complications must be given due consideration before PPI therapy is prescribed.

Possible strategies to mitigate the risk

The established guidelines help physicians identify patients requiring PPI therapy and treatment duration, with the recommendations broadly generalizing which patients can benefit from therapy. Several studies have suggested that deprescribing guidelines must be identified and put into practice [13,15]. In 2017, the Canadian Family Physician published deprescribing guidelines for PPIs. Deprescribing refers to reducing, stopping, or using "as needed" dosing. According to the recommendations, PPIs should be deprescribed in patients who suffer from heartburn and have completed a minimum of four weeks of treatment with resolved symptoms. Patients with Barrett's esophagus, severe esophagitis, or a history of bleeding gastrointestinal ulcers are exempt from these recommendations. They suggested an algorithm to help physicians decide the course of deprescribing PPIs. Alternatives to PPI therapy for occasional symptomatic control included H2 antagonists and non-pharmacological methods [31]. There is a great need to educate patients on the risks of long-term PPIs and for appropriate deprescribing of these medications. The involvement of pharmacists in the instruction of patients and establishment of stewardship programs can improve patient compliance and improve the effectiveness of therapy [32].

Limitations of the Study

Since the topic of PPI side effects is extensive, we focused on the adverse effects seen with clopidogrel interaction and cardiovascular side effects exhibited in new users and GERD patients. We examined many impactful studies that may not have grasped all the available data for evaluation. As several trials and meta-analyses have been conducted on this topic, all relevant data could not be evaluated. Our objectives did not include a detailed discussion of the mechanism of cardiovascular side effects of PPIs as an aim for this study, with further research still required to draw reliable conclusions.

## Conclusions

Numerous studies have been conducted over the years to assess the cardiovascular complications of proton pump inhibitors. Our study found that there is evidence that proton pump inhibitors can be associated with cardiovascular complications. Although the results of many studies vary from showing no significant impact of PPIs to a direct association between PPI and cardiovascular endpoints, there has been no causal relationship identified yet. Further studies must be done beyond meta-analyses to directly assess the impact of PPIs on patients with and without comorbidities. This could include case-control or randomized controlled trials assessing various cardiac endpoints in patients with PPI therapy. The mechanism of action for cardiovascular complications is yet to be confirmed. Our study finds that although guidelines do exist for the prescription of PPIs, implementation of these guidelines is still a challenge, with many unnecessary prophylactic PPI prescriptions occurring globally. Before prescribing, patient screening should be done to include cardiovascular and gastroenterological workups. Physicians must follow up and deprescribe PPIs when the indications are no longer met. Until the relationship between PPIs and cardiovascular complications can be well defined with more data, screening of patients requiring PPI therapy becomes essential, and evaluating the patient profile for comorbidities that may increase risks of complications should be done.
